# Admission vitamin D status is associated with discharge destination in critically ill surgical patients

**DOI:** 10.1186/s13613-015-0065-9

**Published:** 2015-09-17

**Authors:** Karolina Brook, Carlos A. Camargo, Kenneth B. Christopher, Sadeq A. Quraishi

**Affiliations:** Department of Anesthesia, Critical Care, and Pain Medicine, Massachusetts General Hospital, Harvard Medical School, 55 Fruit Street, GRJ 402, Boston, MA 02114 USA; Department of Emergency Medicine, Massachusetts General Hospital, Boston, MA USA; Department of Medicine, Harvard Medical School, Boston, MA USA; Department of Epidemiology, Harvard School of Public Health, Boston, MA USA; Department of Medicine, Brigham and Women’s Hospital, Boston, MA USA; Department of Anaesthesia, Harvard Medical School, Boston, MA USA

**Keywords:** Vitamin D, 25-hydroxyvitamin D, Critical illness, Discharge destination, Patient-centered outcome

## Abstract

**Background:**

Discharge destination after critical illness is increasingly recognized as a valuable patient-centered outcome. Recently, vitamin D status has been shown to be associated with important outcomes such as length of stay (LOS) and mortality in intensive care unit (ICU) patients. Our goal was to investigate whether vitamin D status on ICU admission is associated with discharge destination.

**Methods:**

We performed a retrospective analysis from an ongoing prospective cohort study of vitamin D status in critical illness. Patients were recruited from two surgical ICUs at a single teaching hospital in Boston, Massachusetts. All patients had 25-hydroxyvitamin D (25OHD) levels measured within 24 h of ICU admission. Discharge destination was dichotomized as non-home or home. Locally weighted scatterplot smoothing (LOWESS) was used to graph the relationship between 25OHD levels and discharge destination. To investigate the association between 25OHD level and discharge destination, we performed logistic regression analyses, controlling for age, sex, race, body mass index, socioeconomic status, acute physiology and chronic health evaluation II score, need for emergent vs. non-emergent surgery, vitamin D supplementation status, and hospital LOS.

**Results:**

300 patients comprised the analytic cohort. Mean 25OHD level was 19 (standard deviation 8) ng/mL and 41 % of patients had a non-home discharge destination. LOWESS analysis demonstrated a near-inverse linear relationship between vitamin D status and non-home discharge destination to 25OHD levels around 10 ng/mL, with rapid flattening of the curve between levels of 10 and 20 ng/mL. Overall, 25OHD level at the outset of critical illness was inversely associated with non-home discharge destination (adjusted OR, 0.88; 95 % CI 0.82–0.95). When vitamin D status was dichotomized, patients with 25OHD levels <20 ng/mL had an almost 3-fold risk of a non-home discharge destination (adjusted OR, 2.74; 95 % CI 1.23–6.14) compared to patients with 25OHD levels ≥20 ng/mL.

**Conclusions:**

Our results suggest that vitamin D status may be a modifiable risk factor for non-home discharge destination in surgical ICU patients. Future randomized, controlled trials are needed to determine whether vitamin D supplementation in surgical ICU patients can improve clinical outcomes such as the successful rate of discharge to home after critical illness

## Background

Advances in acute care medicine have resulted in declining mortality rates after critical illness [1–4]. Nonetheless, survival, while evidently a positive outcome, does not truly reflect the health condition of a patient upon discharge from the intensive care unit (ICU). Among survivors, 5–10 % of ICU patients transition to chronic critical illness, with estimates of between 100,000 and 250,000 such patients in the United States at any given point in time [5–8]. These chronically critically ill patients continue to depend on myriad intensive care resources outside of the acute care setting [7–9]. As such, within the context of a healthcare system gravitating towards patient-centered outcomes, the concept of disability-free survival is gaining popularity [10]. In hospitalized patients, discharge destination is a potential early indicator of disability-free survival [11–13], and the ability to predict or identify modifiable risk factors for discharge destination may improve patient-centered health outcomes.

Discharge destination is known to be affected by a variety of factors, which not only arise during the course of hospitalization, but may also be influenced by patient-related factors that pre-date hospital admission. In particular, socioeconomic factors, such as insurance coverage [14], the level of support at home, and baseline functional status [15], have been shown to influence discharge destination. Additional factors, including older age, use of mechanical ventilation or the need for tracheotomy (in the setting of chronic respiratory failure), the presence of severe pressure ulcers, declines in cognitive function, and metabolic or nutritional derangements (e.g., hypoalbuminemia) may influence discharge destination after ICU admission [16–18]. Notably, while many of these identified risk factors are potentially modifiable, easily and/or rapidly correcting them can be a challenge in the clinical setting.

Although vitamin D is typically recognized for its role in promoting optimal musculoskeletal health [19, 20], emerging data suggests that vitamin D status, as determined by measuring its most widely accepted proxy, circulating 25-hydroxyvitamin D (25OHD) [21], may also influence various ICU-related outcomes, including length of stay (LOS), readmission, overall costs, and mortality [22–24]. Moreover, low vitamin D status has an estimated prevalence of 40–80 % among critically ill patients [25–29] and can be rapidly corrected [24, 30, 31]. And while 25OHD levels may influence several provider-centered health outcomes, its role in patient-centered outcomes among survivors of critical illness is unclear. Therefore, the goal of our study was to investigate whether vitamin D status on admission to the ICU is associated with discharge destination in critically ill surgical patients.

## Methods

We performed a retrospective analysis of the data from an ongoing prospective cohort study designed to assess vitamin D status in critically ill patients. A subset of these patients was previously described in studies that investigated the association of vitamin D status with duration of mechanical ventilation and 90-day mortality in ICU patients [22, 32]. For the present study, subjects were recruited from two, 18-bed surgical ICUs at the Massachusetts General Hospital (MGH), in Boston, USA. Both ICUs received admissions from all surgical services except for Cardiac Surgery. All subjects were enrolled between 06/01/2012 and 05/30/2015. MGH is a 1052-bed, teaching hospital and a level-one trauma center, which serves a diverse population in and around Eastern Massachusetts. The Partners Human Research Committee (local Institutional Review Board) approved the study protocol.

### Inclusion and exclusion criteria

All adult males and females, ≥18 years of age, and who were expected to require at least 48 h of critical care (as determined by the treating ICU team), were deemed eligible to participate. Informed consent was obtained either directly from subjects or appropriate healthcare surrogates. Subjects were only included in the study if blood samples to assess vitamin D status could be obtained within 24 h of admission to the ICU. Exclusion criteria included a known history of anemia at the time of ICU admission (defined as hematocrit <25 %), pregnancy or immediate post-partum status, and history of vitamin D supplementation ≥4000 IU/day. To minimize confounding from either partially treated, new-onset illness, or chronic illnesses, subjects were also excluded if they were transferred from another ICU or had been in an ICU within year of the most current admission. Patients expected to transition to “comfort only measures” were also excluded.

### Blood sample processing and biomarker assays

Following informed consent, fresh blood was acquired from an indwelling vascular catheter and was collected directly into an ethylenediaminetetraacetic acid-containing tube (lavender top). The sample was immediately stored on ice and then centrifuged within 30 min to separate out plasma. All samples were centrifuged at 2,300 rpm for 15 min at a temperature of 4 °C. The separated plasma was immediately transferred to polypropylene tubes and stored at −80 °C until biomarker testing was ready to be initiated. Assays were performed at the Harvard Medical School Clinical and Translational Science Award core laboratory at MGH. Plasma 25OHD (combined D_2_ and D_3_) levels were measured by enzyme-linked immunoabsorbent assay (ELISA), using commercially available kits (Abbott Laboratories, Abbott Park, IL, USA). Intra- and inter-assay coefficients of variation were both <10 %.

### Demographic and clinical data collection

The MGH electronic medical records system was used to obtain baseline information related to the following: age, sex, race, body mass index (BMI), socioeconomic status (SES), acute physiology and chronic health evaluation (APACHE) II score, type of surgical patient, vitamin D supplementation status, hospital LOS, and discharge destination. We estimated SES by abstracting the residential zip code from each medical record and cross-referencing it with the US Census Bureau per capita income data [33] specific to each locality. Zip codes in the top third of the per capita income rankings for Massachusetts were considered high SES, middle third were considered moderate SES, and those in the bottom third were considered low SES. Sex, race, type of surgical patient, and in-hospital vitamin D supplementation status were dichotomized as female vs. male, non-white vs. white, emergent vs. non-emergent, and ≤1000 IU vs. >1000 to <4000 IU per day throughout the hospitalization, respectively. Discharge destination was also dichotomized as non-home vs. home. A non-home discharge included all in-hospital deaths, transfers to another healthcare facility for patients who lived at home before ICU admission, and/or transition to a higher level of care compared to baseline (e.g., nursing home resident who was discharged to an acute rehabilitation hospital after ICU admission and acute care on the general wards at MGH). A home discharge included all patients who returned home (with or without home health services) or returned to the same level of care as baseline (e.g., nursing home resident who was discharged back to his/her nursing home after ICU admission and acute care on the general wards at MGH). All other variables were considered as continuous data.

### Locally weighted scatterplot smoothing analysis

Locally weighted scatter plot smoothing (LOWESS) was used to graphically represent the relationship between 25OHD levels and the risk of non-home discharge. LOWESS curves are a form of nonparametric regression, which summarize a relationship between two variables in a fashion that initially relies on limited assumptions about the form or strength of the relationship [34]. The rationale and methods underlying the use of LOWESS for depicting the local relationship between measurements of interest across parts of their ranges have previously been described [35].

### Statistical analysis

Descriptive statistics were calculated for subjects with plasma 25OHD levels <20 ng/mL vs. those with levels ≥20 ng/mL. Continuous data were reported as means with standard deviations (SDs) or medians with interquartile ranges (IQRs). Comparison of characteristics was performed using *t* test and Mann–Whitney analyses for normally distributed variables and nonparametric variables, respectively. Categorical values were expressed as proportions and compared using Chi-squared tests.

Since we considered discharge destination as a dichotomous variable, logistic regression analysis was used to model the relationship between plasma 25OHD levels and discharge destination, while controlling for biologically plausible covariates. In this approach, we controlled for: (1) age; (2) sex; (3) race; (4) BMI; (5) SES; (6) type of surgical patient; (7) APACHE II score; (8) hospital LOS; and (9) vitamin D supplementation. We then repeated the analysis with 25OHD levels dichotomized as <20 vs. ≥20 ng/mL. This threshold was selected based on existing conservative guidelines regarding optimal vitamin D status [36] and observed relationship between 25OHD levels and discharge destination on the LOWESS curve. Results are reported as odds ratios (ORs) with 95 % confidence intervals (CIs).

We performed an a priori sample size calculation using the previously described biologically plausible model for the association between plasma 25OHD levels and discharge destination. Based on previous studies performed by our group and others [37–39] on discharge destination, we assumed a 40 % discharge to home rate in patients with plasma 25OHD levels ≥20 ng/mL and a 20 % discharge to home rate in patients with levels <20 ng/mL. To detect this difference in discharge rate between groups with a power of 0.8 and with alpha set at 0.05, a minimum sample size of 82 patients in each group would be required for the present study. All analyses were performed in STATA 13.0 (StataCorp LP, College Station, TX). A two-tailed *P* < 0.05, or 95 % CI that did not span 1, was considered statistically significant.

## Results

A total of 300 patients comprised the final analytic cohort (Table 1). The mean age was 66 (SD 16) years and most subjects were male (58 %) as well as white (90 %). Overall, mean BMI was 29 (SD 7) kg/m^2^. Approximately 26, 40, and 35 % of patients were from low, moderate, and high SES neighborhoods, respectively, while 14 % of patients had emergency surgery. The mean APACHE II score was 17 (SD 9), median hospital LOS was 9 (IQR 6–14) days, and mean 25OHD level was 19 (SD 8) ng/mL. The overall in-hospital mortality rate was 16 and 41 % of patients had a non-home discharge destination. Among patients who survived to hospital discharge, the non-home destination rate was 30 %.

**Table 1:** Demographic factors, baseline information, and clinical outcomes in surgical intensive care unit patients according to vitamin D status at initiation of care (*n* = 300)

Characteristic	25(OH)D <20 ng/mL (*n* = 186)	25(OH) ≥20 ng/mL (*n* = 114)	*P* value
Age (years)	64 (SD 16)	69 (SD 15)	**<** *0.01*
Sex (%)			0.67
Female	41	44	
Male	59	56	
Race (%)			0.14
Non-white	12	6	
White	88	94	
BMI (kg/m^2^)	29 (SD 8)	28 (SD 6)	0.22
SES			0.09
Low	26	25	
Moderate	39	41	
High	36	33	
Patient type (%)			0.21
Emergent	16	10	
Non-emergent	84	90	
APACHE II	17 (SD 9)	15 (SD 7)	*0.03*
Hospital LOS (days)	9 (IQR 6–15)	9 (IQR 6–12)	0.32
Vitamin D supplementation (%)			**<** *0.01*
<1000 IU/day	88	72	
≥1000 to <4000 IU/day	12	28	
25OHD (ng/mL)	14 (SD 4)	27 (SD 7)	**<** *0.001*
In-hospital mortality (%)			*0.03*
Alive	80	91	
Deceased	20	9	
Discharge destination (%)			**<** *0.01*
Non-home	48	29	
Home	52	71	

*BMI* body mass index, *SES* socioeconomic status, *APACHE II* acute physiology and chronic health evaluation II, *LOS* length of stay, *25OHD* 25-hydroxyvitamin D

Data presented as either mean with standard deviation (SD), median with interquartile range (IQR), or proportions and compared using *t* tests, Mann–Whitney tests, and Chi-squared tests, respectively. Significant *P* values (<0.05) are shown in italic. To convert ng/mL to nmol/L, please multiply by 2.496

**Fig. 1:**
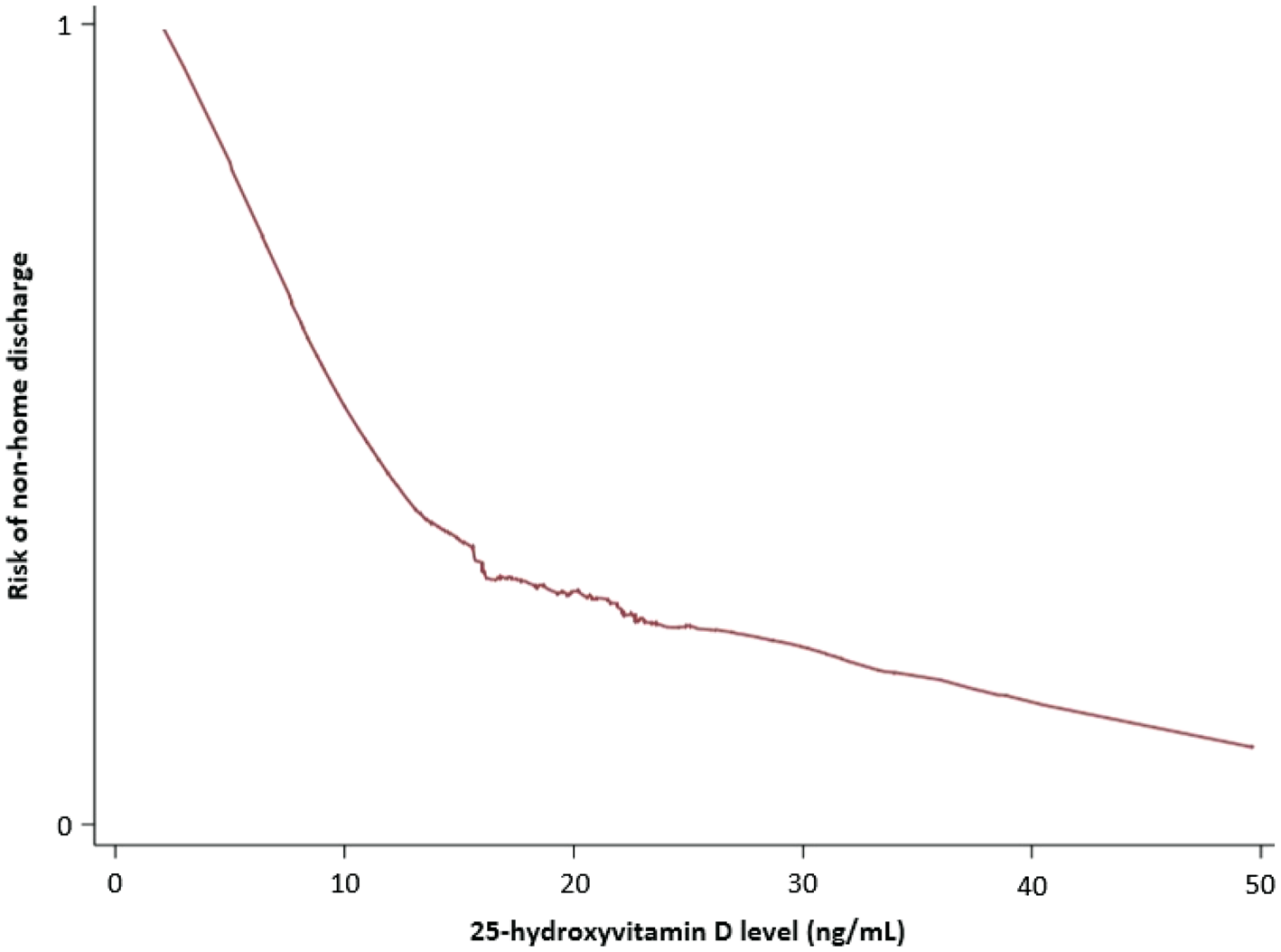
LOWESS curve analysis demonstrates the relationship between vitamin D status and the risk of non-home discharge destination for 25OHD levels of 0–50 ng/mL

LOWESS curve analysis (Fig. 1) demonstrated a near-inverse linear relationship between vitamin D status and the risk of non-home discharge destination up to 25OHD levels of around 10 ng/mL. Between 25OHD levels of 10 and 20 ng/mL, there was significant flattening of the curve. Beyond 25OHD levels of 20 ng/mL, the risk curve continued to flatten incrementally. Logistic regression analysis (Table 2), while controlling for biologically plausible covariates, demonstrated an inverse association between admission plasma 25OHD levels and non-home discharge destination (OR per 1 ng/mL, 0.88; 95 % CI 0.82–0.95). When vitamin D status was considered as a dichotomous variable, patients with 25OHD <20 ng/mL were more than 2.5 times more likely to have a non-home discharge destination compared to patients with 25OHD ≥20 ng/mL (OR, 2.74; 95 % CI 1.23–6.14). Additional variables found to be independently associated with non-home discharge destination in these models were APACHE II score, hospital LOS, and vitamin D supplementation.

**Table 2:** Biologically plausible models to test the association of admission 25-hydroxyvitamin D level with non-home discharge destination in surgical intensive care unit patients (*n* = 300)

Covariate	OR (95 % CI) 25OHD (continuous variable)	OR (95 % CI) 25OHD (<20 vs. ≥20 ng/mL)
Age (years)	1.01 (0.98–1.04)	1.00 (0.98–1.03)
Sex		
Female	–	–
Male	0.81 (0.36–1.83)	1.08 (0.51–2.31)
Race		
Non-white	–	–
White	1.57 (0.96–2.56)	2.12 (0.52–8.68)
BMI (kg/m^2^)	2.07 (0.58–8.47)	0.97 (0.91–1.02)
SES		
Low	–	–
Moderate	1.09 (0.38–3.09)	1.08 (0.39–3.02)
High	1.07 (0.36–3.18)	0.92 (0.32–2.65)
Patient type		
Emergent	–	–
Non-emergent	1.73 (0.32–9.32)	1.66 (0.32–8.66)
APACHE II	*1.33 (1.22–1.45)*	*1.32 (1.22–1.43)*
Hospital LOS (days)	*1.18 (1.11–1.25)*	*1.18 (1.11–1.25)*
Vitamin D supplementation (IU/day)		
<1000	–	–
≥1000 to <4000	0.56 (0.17–1.83)	*0.23 (0.08–0.67)*
25OHD (ng/mL)	*0.88 (0.82–0.95)*	*2.74 (1.23–6.14)*

(*–*) represents each referent variable

Statistically significant variables are highlighted in italic

*OR* odds ratio, *CI* confidence interval, *BMI* body mass index, *SES* socioeconomic status, *APACHE II* acute physiology and chronic health evaluation II, *LOS* length of stay, *25OHD* 25-hydroxyvitamin D

## Discussion

In this retrospective cohort study, we investigated whether vitamin D status determined at initiation of critical care was associated with subsequent discharge destination from the hospital. We demonstrated that plasma 25OHD levels measured on admission to the surgical ICU were inversely associated with the risk of a non-home discharge destination (i.e., patients with low 25OHD levels are more likely to have a non-home discharge destination). This relationship was most prominent when comparing patients with 25OHD levels <20 ng/mL to those with levels ≥20 ng/mL. However, due to the observational nature of this study, a causal inference about the effect of vitamin D status on discharge destination in critically ill surgical patients is limited. And while the association between vitamin D status and various provider-centered outcomes, such as hospital LOS and readmission rates, has previously been reported [22–24], its impact on a patient-centered outcome, such as discharge destination after hospitalization, has not been well explored. Nonetheless, the biologic plausibility of the relationship between vitamin D status and discharge destination is undeniable.

The wide-ranging role of vitamin D in musculoskeletal health, epithelial barrier site integrity, and regulation of both innate as well as adaptive immunity, in addition to calcium homeostasis, may explain how optimizing 25OHD levels in critically ill patients may improve outcomes [40]. Proximal muscle group weakness and atrophy, particularly of type II fibers, are associated with low vitamin D status [41–43], and profound deconditioning associated with muscle wasting in the ICU is associated with long-term physical as well as functional disability [44–46]. Vitamin D metabolites have been shown to affect muscle metabolism through a variety of pathways [43]. Most importantly, the vitamin D membrane receptor (VDR), which binds the most biologically active vitamin D metabolite 1,25-dihydroxyvitamin D (1,25OHD), is expressed in human skeletal muscle cells [47, 48]. Modulated both by genetic transcription after transportation to the nucleus [49], and by direct interaction with muscle cell membranes resulting in second-messenger pathways [50], the 1,25OHD–VDR interaction results in increased calcium uptake within muscle cells [51].

Additionally, vitamin D has been shown to be important for the maintenance of epithelial and mucosal cells, in particular preserving the barrier functions of these cells [52]. Activity of the 1-α-hydroxylase that converts 25OHD–1,25OHD has been demonstrated in epithelial cells of the respiratory system [53], intestine [54, 55], skin [56, 57], and endothelium [58]. Vitamin D supplementation has been shown to increase the growth and differentiation of respiratory epithelial cells [59], while low vitamin D status has been shown to cause pulmonary epithelial dysfunction in mice [60]. Vitamin D metabolites have also been shown to promote colonic epithelial barrier integrity and function through the preservation of intercellular tight junctions in both in vitro and murine studies [61, 62], with low vitamin D status increasing the risk of colitis in animal models [62]. Furthermore, keratinocytes not only produce and metabolize the vitamin D precursor [63], but keratinocyte differentiation is in turn modulated by vitamin D and is essential for maintaining healthy skin [64, 65]. And finally, in vitro studies have shown that in endothelium, supplementing with vitamin D reduces the generation of anion superoxides, prevents free radical release [66], and promotes endothelial cell viability through the production of nitric oxide [66, 67]. Therefore, compromised natural barriers in the setting of low vitamin D status likely increases susceptibility to nosocomial infections, including central line-associated blood stream infections, hospital-acquired pneumonia, and *Clostridium difficile* infections [40, 68, 69], which all not only increase the risk of mortality, but also lead to prolonged hospitalization and likely compound the functional decline associated with critical illness.

VDR is also expressed in cells of the innate and adaptive immune system [70], including T cells, activated B cells, and dendritic cells [71–75]. Low vitamin D status is linked to the Toll-like receptor stimulation of macrophages that leads to increased expression of VDR [76], assists with conversion of 25OHD–1,25OHD [77], and also upregulates the expression of endogenous antimicrobial peptides, cathelicidin (LL-37) and β-defensin [20, 76, 78]. These peptides have known activity against Gram-positive and Gram-negative bacteria, mycobacteria, viruses, and fungi [79], thereby suggesting an important role of optimal vitamin D status in prevention and potentially treatment of serious infections [80]. Indeed, recent evidence suggests that high-dose cholecalciferol (vitamin D3) supplementation in patients with severe sepsis or septic shock increases systemic cathelicidin levels, while modulating immunoregulatory cytokines such as interleukin (IL)-1β and IL-6 [31]. Previous studies have also suggested that vitamin D is a key regulator of inflammatory cytokines [81–83].

While our findings are intriguing, it is important to discuss the limitations of our study. We performed a retrospective, observational analysis, and therefore, causality cannot be inferred. And despite our efforts to adjust for multiple covariates, the presence of residual confounding that may have contributed to the observed outcomes cannot be ruled out. Indeed, we are unable to fully adjust for baseline functional status, exposure to sunlight, as well as nutritional status before and through hospitalization. Additionally, hospital LOS is often influenced by several factors, such as social and financial issues, that we could not fully adjust for in our model. Of note, we elected to use hospital LOS instead of ICU LOS, since patients may often remain in the ICU due to a lack of available beds on the general wards. Furthermore, discharge destination may be affected by financial and insurance considerations, the availability of home help, and family/social support—all factors that we are unable to adjust for in the present study. Moreover, we only recruited a limited number of patients from two surgical ICUs at a single, large referral center, which may decrease the generalizability of our results to all ICU patients. For the most part, patients recruited into the study were also racially homogenous, and this limited diversity may further affect the generalizability of our findings. In the present study, only 25OHD levels assessed within 24 h of ICU admission were considered; it is possible that this single data point does not reflect the variation in vitamin D status with inflammation, metabolic changes, fluid shifts, and systemic micro and macronutrient needs for the entire duration of critical illness [22, 84].

It is worth noting that more patients in our analytic cohort with admission 25OHD levels ≥20 ng/mL received vitamin D supplementation between ≥1000 and <4000 IU/day during hospitalization, as compared to patients with levels <20 ng/mL (28 vs. 12 %, respectively; *P* < 0.01). This is due to the fact that patients with higher initial 25OHD levels were more likely to be taking vitamin D supplementation before hospitalization and as a result, it was more likely to be continued after ICU admission. Measurement of vitamin D status is not part of routine care in our surgical ICUs and all admission 25OHD levels were measured from frozen plasma samples several weeks after hospitalization (and therefore not temporally related to clinical decision-making). Since only 18 % of the entire study cohort received vitamin D supplementation during hospitalization, we were likely underpowered to detect an independent association between supplementation status and discharge destination (the primary outcome) when the exposure of interest in our regression model (i.e., 25OHD) was considered as a continuous variable. Dichotomizing the exposure (<20 vs. ≥20 ng/mL) improved the ability of our model to discriminate between patients who were and were not discharged home, and in doing so, there was likely sufficient power to also detect an independent association of supplementation status with discharge destination. Notable also, was the fact that the proportion of patients in each SES group did not differ by vitamin D status. This may be related to the generally high prevalence of low vitamin D status in New England (due to geography and weather) and the enrollment of patients more likely to be indoors during the day (e.g., elderly, retirees, office workers). As such, these issues will need to be addressed in larger future studies to replicate and extend the results of our current study.

## Conclusion

Our results suggest that vitamin D status may be a modifiable risk factor for discharge destination in surgical ICU patients. Given that suboptimal 25OHD levels are highly prevalent in ICU patients [25–29], further prospective studies are needed to validate our findings, to assess the potential benefits of optimizing 25OHD levels in critical illness, and to identify the mechanism by which vitamin D may impact patient-oriented outcomes in ICU patients.

## Abbreviations

LOS: length of stay
ICU: intensive care unit
25OHD: 25-hydroxyvitamin D
LOWESS: locally weighted scatterplot smoothing
MGH: Massachusetts General Hospital
ELISA: enzyme-linked immunoabsorbent assay
BMI: body mass index
SES: socioeconomic status
APACHE II: acute physiology and chronic health evaluation
SD: standard deviation
IQR: interquartile range
OR: odds ratio
CI: confidence interval
VDR: vitamin D membrane receptor
1,25OHD: 1,25-dihydroxyvitamin D
IL: interleukin
